# O4.3. INCREASED CEREBRAL BLOOD FLOW AFTER SINGLE DOSE OF ANTIPSYCHOTICS IN HEALTHY SUBJECTS DEPENDS ON DOPAMINE D2 RECEPTOR DENSITY PROFILES EVALUATED WITH PET AND MRNA EXPRESSION DATA.

**DOI:** 10.1093/schbul/sby015.209

**Published:** 2018-04-01

**Authors:** Pierluigi Selvaggi, Mattia Veronese, Peter CT Hawkins, Ottavia Dipasquale, Gaia Rizzo, Alessandro Bertolino, Juergen Dukart, Fabio Sambataro, Steven CR Williams, Federico Turkheimer, Mitul Mehta

**Affiliations:** 1 Institute of Psychiatry, Psychology and Neuroscience, King’s College London; 2 Imanova Ltd., Centre for Imaging Sciences, Hammersmith Hospital; 3 University of Bari ‘Aldo Moro’; 4 Translational Medicine Neuroscience and Biomarkers, F. Hoffmann-La Roche Ltd, Basel, Switzerland; 5 University of Udine

## Abstract

**Background:**

Previous studies measuring cerebral blood flow (CBF) with magnetic resonance sequences like Arterial Spin Labelling (ASL) showed that patients with schizophrenia (SCZ) have increased CBF in basal ganglia and reduced blood flow in cortical areas like the prefrontal cortex. It is still not clear whether these abnormalities are related to antipsychotic treatment or rather they reflect a disease trait independent from medication. Interestingly, administration of single dose of antipsychotics in healthy volunteers produce marked functional effects that are in the same region reported as altered in SCZ. These effects are thought to depend on dopamine D2 receptor (D2R) blockade, although their relationship with antipsychotic pharmacodynamics has not been fully established yet. In fact, the haemodynamic nature of CBF measures makes difficult to interpret drug effects in terms of altered neurotransmission function. Here, we tested whether CBF changes induced by different antipsychotics mirror receptor distribution profiles of D2R. We evaluated the correlation of CBF variation with receptor density as measured with PET and brain mRNA expression extracted from the Allen Human Brain Atlas (ABA).

**Methods:**

Forty-two healthy male subjects were enrolled in a double blind, randomized, placebo-controlled, crossover study. Participants were randomized in two equal parallel groups to receive a single dose of antipsychotic/placebo in three separate sessions. In Group 1 placebo, olanzapine 7.5mg (OLA) or haloperidol 3mg (HAL) were administered before the MRI scan. In Group 2 participants received placebo, 0.5mg (lowRIS) or 2mg (highRIS) of risperidone. Regional CBF was assessed with pseudo-continuous ASL (pCASL) sequence. For each antipsychotic, a paired T-test was performed in SPM12 with global CBF values as covariate of no interest.

A template image of dopamine D2 receptor density was derived from 6 PET scans in healthy volunteers using the high affinity D2/D3 antagonist ligand [18F]-Fallypride. Brain mRNA expression values for DRD2 gene (coding for D2R) were extracted from the ABA dataset by using the MENGA toolbox. CBF contrast images and the [18F]Fallypride BPND template were segmented into 83 ROIs by using the Desikan-Killiany Atlas. The regional changes in CBF against placebo (∆CBF) were compared with regional BPND values and gene expression maps using multivariate correlations.

**Results:**

For all antipsychotics, CBF changes in each ROI were directly proportional to [18F]Fallypride non displaceable binding potential (BPND) values (OLA R2= 0.24, HAL R2= 0.61, lowRIS R2= 0.54, highRIS R2= 0.52, all p<0.001) and DRD2 mRNA expression levels (OLA R2= 0.04, HAL R2= 0.15, lowRIS R2= 0.19, highRIS R2= 0.20, all chance likelihood <2%).

**Discussion:**

In the present study, we were able to show that the CBF increase induced by antipsychotic is directly proportional to D2R concentration in the brain, as indexed by PET BPND maps and mRNA expression levels. Interestingly, the association strength between ∆CBF and brain receptor distribution profiles mirrored differential D2R affinity between the tested drugs. Overall, these results indicate that CBF increases after administration of a single dose of antipsychotics actually reflect known pharmacodynamics profile of these compounds. In addition, these results further reinforce previous evidence suggesting the role of D2R blockade as a mechanism behind increased CBF induced by antipsychotics. Finally, CBF is ultimately a functional marker and this work is important in bridging the considerable gap between the pharmacokinetic and pharmacodynamic effects of compounds with unclear brain functional effects like antipsychotics.

